# Visualizing Enhanced Microfluidic Electromembrane Desalination Using Nafion-Coated Heterogeneous Ion-Exchange Membranes

**DOI:** 10.3390/molecules31040719

**Published:** 2026-02-19

**Authors:** Hyunwoo Choi, Bonseung Ku, Seokhee Han, Bumjoo Kim

**Affiliations:** 1 Department of Mechanical and Automotive Engineering, Kongju National University, Cheonan 31080, Republic of Korea; wkrrkwjdtls@naver.com (H.C.); koo2111@naver.com (B.K.); 2 Department of Future Convergence Engineering, Kongju National University, Cheonan 31080, Republic of Korea; han991001@naver.com

**Keywords:** heterogeneous ion-exchange membrane, electro-convection, surface modification, water splitting, visualization, Nafion coating, desalination, over-limiting current, microfluidics

## Abstract

Heterogeneous ion-exchange membranes (IEMs) are cost-effective but suffer from low electrochemical efficiency due to surface inhomogeneities. While surface coating with homogeneous ionomers is a known modification strategy, its direct impact on electro-hydrodynamic behavior and desalination performance has rarely been visually verified. In this study, we employed a microfluidic platform to visualize and quantify the performance enhancement of Nafion-coated heterogeneous cation exchange membranes (CEMs). Contrary to conventional theories linking electro-convection (EC) to surface hydrophobicity, our results show that the hydrophilic Nafion coating significantly amplifies EC vortices. Direct visualization revealed that the coating layer acts as an electrical nozzle, inducing intense electric field focusing that triggers macroscopic vortex growth. Furthermore, we visually confirmed that the coating layer physically seals catalytic sites, effectively suppressing parasitic water-splitting reactions. In continuous desalination experiments, this hydrodynamic synergy led to a 32% increase in current efficiency (*CE*: 1.23) and an 18% increase in salt removal ratio (*SRR*: 79.4%) compared to bare membranes in the over-limiting regime. These findings demonstrate that inducing controlled hydrodynamic instability via surface modification is a dominant factor for high-efficiency desalination.

## 1. Introduction

Ion-exchange membranes (IEMs) are critical materials widely utilized across various fields due to their inherent perm-selectivity. Their applications range from mass separation processes like electrodialysis (ED) [1,2,3,4,5,6] to energy conversion and storage systems such as fuel cells, redox flow batteries, and reverse electrodialysis (RED) [7,8], as well as chemical synthesis processes including chlor-alkali and acid-base production [9,10]. Generally, IEMs are classified into homogeneous and heterogeneous membranes based on their manufacturing methods and the fraction of the conductive surface area [1,11,12,13,14]. While homogeneous membranes offer superior electrochemical performance due to their fully conductive surface, they are often prohibitively expensive. In contrast, heterogeneous membranes, despite their higher electrical resistance caused by the presence of non-conductive binders, are valued for their excellent mechanical strength and cost-competitiveness [11,12,13,15]. Regardless of the membrane type, all IEMs experience ion depletion and concentration polarization (CP) at the membrane interface, which fundamentally limits the process performance to the limiting current density (LCD) [1]. Therefore, to achieve much higher salt removal, operation in the over-limiting current (OLC) regime is essential [16,17]. In this regime, the dominant mechanism driving mass transfer is electro-convection (EC), which arises from hydrodynamic instability at the membrane surface [16,18]. However, heterogeneous IEMs face a specific challenge known as the funneling effect, where current is locally concentrated due to the mixed presence of conductive particles and non-conductive binders. Rubinstein et al. [18] theoretically proposed that this surface heterogeneity distorts current paths and induces localized electric field concentration, thereby accelerating concentration polarization.

To overcome the structural limitations of heterogeneous IEMs, such as non-uniform current distribution and low efficiency [11,13], recent studies have actively explored surface modification techniques, specifically coating the membrane surface with homogeneous ionomer solutions like Nafion or sulfonated polymers (MF-4SK) [8,19,20,21,22,23]. Recent studies have increasingly focused on manipulating electro-convective vortices via surface geometric structuring, such as membrane corrugation [24] and geometric modifications [25], to enhance ion transport. Concurrently, understanding the distinct over-limiting mechanisms in heterogeneous membranes compared to homogeneous ones remains a critical challenge for next-generation desalination systems [26]. The mechanism by which such surface modification enhances electrochemical performance has been established through three main perspectives. First is the homogenization of current distribution. Rubinstein et al. [18] proposed a theoretical model suggesting that introducing a conductive homogeneous layer onto a heterogeneous surface optimizes the current line distribution. Gil et al. [27] experimentally verified that the coating layer seals the rough surface of the heterogeneous substrate, redistributing current lines evenly across the entire surface. This mitigates the localized funneling effect, delays the onset of CP, and consequently increases the LCD. Crucially, the coating prevents excessive current localization while maintaining or optimizing the tangential electric field component essential for EC generation. Second is the suppression of water splitting. Typically, specific functional groups or structural defects on the heterogeneous IEM surface act as catalysts that promote water-splitting reactions in the limiting current regime [1]. The generated H^+^ and OH^−^ ions reduce the electric field at the membrane surface, thereby diminishing the driving force for EC. Studies by Pismenskaya et al. [28,29] and Sharafan et al. [30] demonstrated that the homogeneous coating layer blocks these active sites, significantly reducing the water-splitting rate. Andreeva et al. [31] further explained a virtuous cycle where the homogenized current distribution mitigates concentration polarization, while the enhanced EC maintains ion concentration at the surface, further suppressing water splitting. Third is the increase in surface hydrophobicity and EC enhancement. This is the most dominant theory explaining performance improvement in the OLC regime. Belashova et al. [32] observed that the contact angle of heterogeneous IEMs increased significantly from 55° to 64° after coating the surface with a thin film of Nafion. Researchers concluded that this hydrophobic surface reduces friction with the fluid and induces slip, which is a key factor in amplifying hydrodynamic instability within the space charge layer (SCL). The enhanced EC vigorously stirs the solution, reducing the diffusion boundary layer thickness and increasing the mass transfer rate in the OLC regime [1]. In summary, existing literature consistently reports that homogeneous layer coating leads to surface homogenization and increased hydrophobicity, which in turn suppresses water splitting and enhances EC, ultimately improving mass transfer performance.

**Figure 1:**
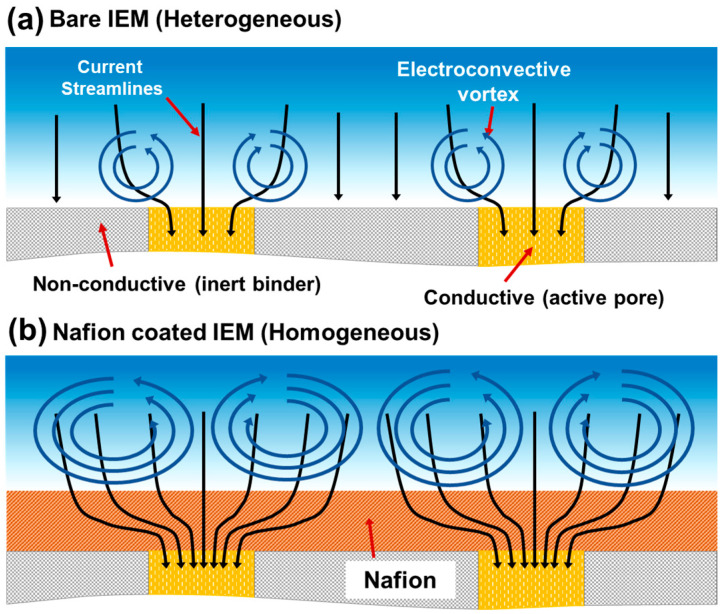
Schematic illustration comparing the current streamline distribution and electro-convective vortex formation at the membrane-solution interface. (**a**) Bare IEM (Heterogeneous): The ionic current is scattered by the non-conductive binders, resulting in weak, localized, and irregular vortices. (**b**) Nafion Coated IEM (Homogeneous): The introduction of a Nafion layer creates a homogeneous conductive surface. As depicted, the coating induces a “constricted funneling” effect where current streamlines converge into the pores, creating a localized high electric field (resistive bottleneck). This geometry significantly amplifies the electro-hydrodynamic instability, resulting in the formation of larger, more robust, and coherent vortices (larger blue spirals) that enhance mass transfer.

Despite these advances, previous studies have largely relied on indirect electrochemical indicators, such as the inflection points in Current-Voltage (I–V) curves or transition time measurements, to explain the EC enhancement effect by coating layers [27,29,31,32,33]. While pioneering works by Kwak et al. [34,35], Jang et al. [36], and Choi et al. [37] have utilized microfluidic devices to visualize EC behavior, these observations were primarily limited to ideal homogeneous membrane systems or simplified geometries. To date, there is a distinct lack of research that directly visualizes and elucidates how the combined effects of water-splitting suppression and surface property changes alter the actual hydrodynamics when Nafion coating is applied to commercial heterogeneous IEMs used in industrial applications (Figure 1). Most critically, no study has yet reported empirical data quantitatively verifying the practical desalination performance in a continuous electrodialysis system using coated heterogeneous IEMs. In other words, while physical theories suggesting that surface modification alters hydrodynamic behavior exist, empirical evidence linking these changes directly to final process efficiency is absent. Therefore, this study aims to go beyond electrochemical analysis by performing real-time visualization of EC formation, growth, and water-splitting phenomena on Nafion-coated heterogeneous IEM surfaces using fluorescent particles and pH indicators within a microfluidic system. Through this, we visually demonstrate the mechanism by which the coating layer suppresses the chronic issue of water splitting in heterogeneous membranes and controls EC instability. Furthermore, by conducting continuous desalination experiments, we quantitatively verify how the altered hydrodynamic characteristics induced by the coating layer impact the final desalination efficiency.

## 2. Results and Discussion

### 2.1. Surface Characterization of Nafion-Coated Heterogeneous Membranes

**Figure 2:**
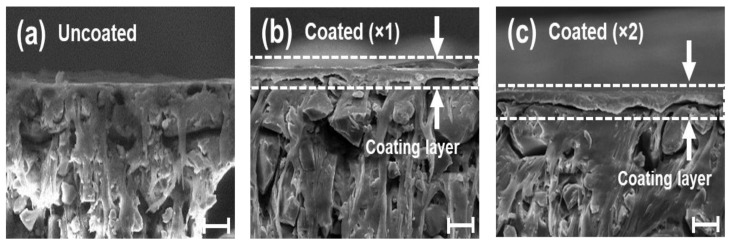
Cross-sectional Scanning Electron Microscopy (SEM) images comparing the microstructural morphology of the base and modified membranes. (**a**) Uncoated: The pristine heterogeneous membrane exhibits a rough and porous structure characteristic of its composite nature, where ion-exchange resin particles are irregularly distributed within the inert binder, leading to surface voids. (**b**) Coated (×1) and (**c**) Coated (×2): The Nafion spin-coating effectively fills the microscopic irregularities and planarizes the surface, forming a dense and homogeneous ionomer layer on top of the substrate. The coating layers, indicated by the white arrows and dashed lines, show thicknesses of approximately 8 µm and 10 µm, respectively. Notably, the interface demonstrates excellent physical adhesion without any signs of delamination, ensuring structural integrity. Scale bars indicate 20 µm.

The cross-sectional microstructures of the bare and Nafion-modified heterogeneous ion-exchange membranes (IEMs) were analyzed using Scanning Electron Microscopy (SEM), as shown in Figure 2. The bare heterogeneous membrane, due to its inherent manufacturing process involving the mixing of ion-exchange resin particles with an inert polymer binder, exhibits a rough surface morphology characterized by numerous voids and discontinuous boundaries between the phases [38]. Such structural heterogeneity can induce localized imbalances in current density and physically disturb the fluid flow at the interface. In contrast, the SEM images of the spin-coated samples (Coated ×1 and ×2) reveal that the Nafion solution effectively infiltrated and leveled the microscopic irregularities and pores of the substrate, resulting in the formation of a highly smooth and homogeneous ionomer layer. Notably, the coating layer demonstrated robust interfacial adhesion with the base membrane without any signs of delamination. This suggests that a physical interlocking mechanism occurred between the coating layer and the substrate during the solvent evaporation process. The thickness of the coating layer was precisely controlled, measuring approximately 8 µm for a single coating and increasing to 10 µm for a double coating, as determined by cross-sectional SEM analysis (Table 1). These results demonstrate that the spin-coating method can effectively mitigate the intrinsic structural defects of heterogeneous membranes and achieve significant surface planarization.

A critical and distinct observation in this study is the alteration of surface wettability. Previous studies focusing on macroscopic electrodialysis systems [27,31,32] have generally reported that modifying the membrane surface to be hydrophobic enhances electro-convection (EC) by promoting hydrodynamic slip. However, the contact angle measurements in this study indicate a contrary trend: the contact angle decreased from 71° in the bare state to approximately 59° after coating, indicating a shift towards a more hydrophilic surface (Table 1). This phenomenon is attributed to the surface reconstruction during the spin-coating and subsequent annealing processes, where the hydrophilic sulfonic acid groups (SO_3_^−^) of the Nafion polymer likely underwent thermodynamic reorientation towards the surface, while the hydrophobic backbone receded. These findings present a significant deviation from conventional theories [16] that primarily link enhanced EC to increased surface hydrophobicity. Instead, our results suggest that factors other than hydrophobicity—specifically, the surface homogeneity and uniform charge distribution—play a pivotal role in the electrochemical behavior observed in this system.

### 2.2. Electrochemical Characteristics

**Figure 3:**
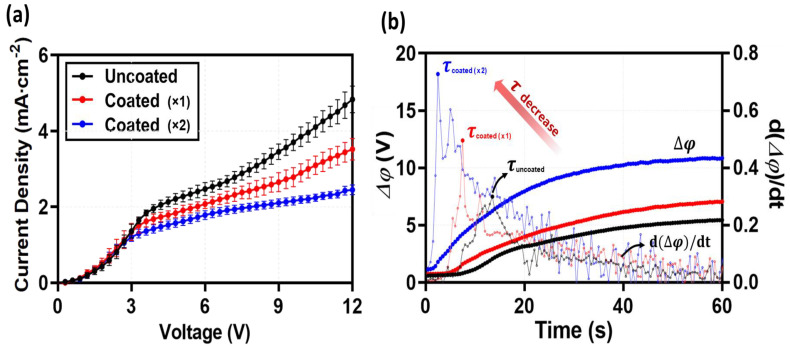
Electrochemical characterization of the ion-exchange membrane system in the microfluidic device. (**a**) Current-voltage (I–V) curves measured at a flow rate of 1 mm/s. While the initial ohmic slopes are comparable, a distinct reduction in the limiting current density (LCD) and over-limiting current is observed as the Nafion coating thickness increases. This trend stands in contrast to macroscopic behaviors and is attributed to the geometric constriction, where the confined swelling of the coating layer creates a resistive bottleneck via the ‘constricted funneling’ effect. (**b**) Chronopotentiometry curves (solid line vs. dashed line) and their time derivatives (dashed lines) under a constant applied current. The transition time (*τ*), marked by the inflection point where the voltage rises sharply due to ion depletion, decreases significantly from the Uncoated to the Coated) membranes (indicated by the red arrow). This rapid onset of concentration polarization further corroborates the formation of a resistive barrier caused by the physical confinement within the microfluidic device.

To evaluate the impact of the coating layer on ion transport characteristics, current-voltage (I–V) curves and chronopotentiometry were analyzed (Figure 3). Generally, previous studies involving the coating of homogeneous ionomers onto heterogeneous IEMs in macroscopic setups have reported enhanced electrochemical performance [18,27,29,31,32,33]. Rubinstein et al. [18] and Mareev et al. [39] demonstrated that such coatings mitigate the funneling effect by redistributing current lines, thereby increasing the LCD and shortening the plateau region. Furthermore, chronopotentiometry typically shows a delayed transition time for coated membranes, attributed to the promotion of equilibrium electro-convection [18,27,29]. However, the results obtained in this microfluidic study exhibit a trend contrary to these conventional macroscopic findings. Analysis of the Ohmic region in the I-V curves (Figure 3a) reveals that the slopes remained comparable regardless of the coating, indicating that the thin coating layer itself did not significantly alter the bulk electrical resistance. In contrast, a distinct reduction in the LCD was observed as the number of coating layers increased. This trend is further supported by the chronopotentiometry analysis (Figure 3b), where the transition time (*τ*), indicating the onset of concentration polarization, decreased drastically from 14 s (Uncoated) to 8 s (Coated ×1) and 3 s (Coated ×2).

This apparent discrepancy implies a fundamental difference between the material properties observed in the free-standing state (Table 1) and the actual operating conditions within the device (in situ). As shown in Table 1, the Nafion coating layer exhibits significant swelling and thickness expansion when hydrated in an unconstrained environment (ex situ). However, within the microfluidic device, the membrane is subjected to tight physical clamping by the PDMS blocks. Consequently, the inherent tendency of the Nafion layer to swell generates significant internal swelling pressure against the rigid channel walls. In this state of confined swelling, the resulting compressive stress leads to a reduction in free volume and the collapse of ionic pathways (pores). Therefore, instead of facilitating ion transport as observed in free-swelling macro-systems, the coating layer in this confined environment functions as a resistive barrier, reducing the effective cross-sectional area for ion flux. This restriction facilitates the early onset of concentration polarization, thereby distinctly reducing the limiting current density (LCD) [7,40,41]. While this reduction in the LCD may appear as a performance degradation, it has significant implications from an electro-hydrodynamic perspective. The forced passage of ionic current through these constricted, high-resistance pathways (constricted pores) induces a steep voltage drop and a consequent electric field focusing effect. This implies that the coating layer does not merely act as a resistor but creates a localized environment with high electric field intensity. This distinctive electrical condition is expected to serve as a critical trigger for the unique fluid dynamics observed in the over-limiting regime, which will be discussed in the following section.

### 2.3. Direct Visualization

**Figure 4:**
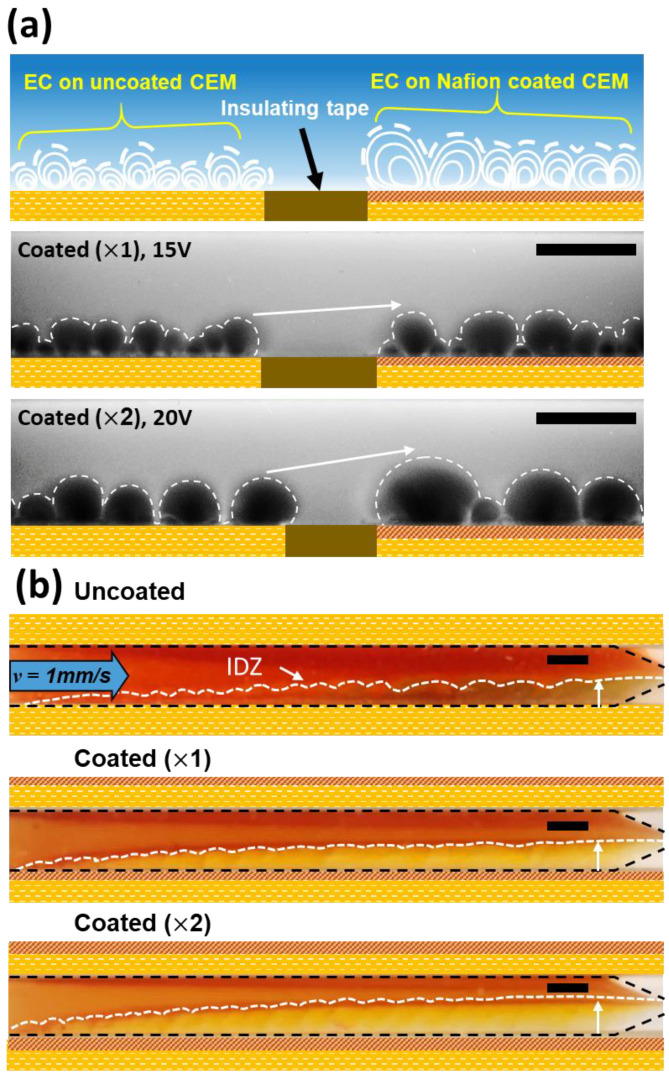
Direct visualization of electro-hydrodynamic instability and interfacial phenomena. (**a**) Simultaneous visualization of electro-convective (EC) vortices on uncoated (**left**) and Nafion-coated (**right**) regions separated by an insulating tape to ensure identical operating conditions. While the uncoated region shows weak, localized micro-vortices, the coated region exhibits robust, macroscopic vortices. This amplification empirically verifies the electrical nozzle effect triggered by current focusing. (**b**) Visualization of the Ion Depletion Zone (IDZ) and pH variations at a constant current of 300 µA (*J* = 7.5 mA/cm^2^). The coated membranes display a significantly stabilized and thickened IDZ (indicated by vertical arrows) compared to the fluctuating interface of the uncoated membrane, demonstrating that the enhanced vortices refresh the boundary layer while the coating physically seals catalytic sites to suppress water splitting. All scale bars indicate 1 mm.

Figure 4 illustrates the evolution of electro-convection (EC) and water splitting behaviors dependent on the number of Nafion coating layers. To elucidate how the increased electrical resistance identified in Section 2.2 translates into hydrodynamic instability, direct visualization was performed using fluorescent particle tracking and pH indicators. Conventional studies introducing homogeneous layers onto IEMs [29,31,33] have typically attributed enhanced EC to increased surface hydrophobicity, which promotes hydrodynamic slip. However, in this study, despite a reduction in hydrophobicity (Table 1), the Nafion-coated membranes exhibited significantly larger and more robust vortices compared to the heterogeneous IEM (Figure 4a). While vortices on the uncoated membrane remained irregular and localized, those on the coated membranes evolved into macroscopic circulating flows dominating the entire channel height. This paradoxical phenomenon is interpreted as the result of electric field focusing induced by resistive confinement. The coating layer, with its restricted swelling, acts as an “electrical nozzle”. Specifically, the coating layer collects ions from the non-conductive binder regions and funnels them into the active pores (geometric funneling). However, due to the restricted swelling, this concentrated ion flux encounters a highly densified coating matrix at the pore entrance. This ‘constricted funneling’ creates a resistive bottleneck that reduces the limiting current density (LCD). The concentration of current through these constrictions generates intense localized electric fields, serving as a powerful trigger for electro-convective instability as described by Rubinstein et al. [18]. Although our surface is physically smooth, this mechanism is electro-hydrodynamically analogous to the enhancement of EC observed in membranes with engineered surface microreliefs or geometric patterns [36,42,43,44], where localized field distortions accelerate fluid motion.

The formation of the Ion Depletion Zone (IDZ) was visualized by monitoring the optical intensity variations of the pH indicator dye. Based on the correlation between dye intensity and local concentration [34], the IDZ boundary was determined as the region where the optical intensity (color gradient) shifted significantly relative to the bulk solution. Consequently, in the pH visualization (Figure 4b), the uncoated heterogeneous IEM exhibited irregular color gradients along the interface, indicating local pH fluctuations and unstable ion depletion zones (IDZ). This suggests the occurrence of irregular water splitting reactions at exposed structural defects and catalytic sites on the heterogeneous surface [1,12]. In contrast, the Nafion-coated membranes showed suppressed local color variations and the formation of a much thicker and more stable IDZ. A critical observation is the correlation between vortex size and IDZ thickness. As the coating layers increased, the earlier onset and intensification of vortices corresponded to a substantial expansion of the IDZ. This indicates a dual effect of the homogeneous Nafion layer: it physically seals the catalytic sites (specifically, the structural defects at the interfaces between the conductive resin particles and the non-conductive binder) to suppress water splitting [28,37,45], while the enhanced EC vortices act as powerful mixers, rapidly transporting fresh electrolyte to the membrane surface. Consequently, the thickened IDZ observed in the coated membranes represents not a region of stagnation, but an extended instability region where mass transfer is maximized by vigorous electro-convection.

### 2.4. Desalination Performance

To quantitatively evaluate the desalination performance, the Salt Removal Ratio (*SRR*), Current Efficiency (*CE*), and Energy Per Ion Removal (*EPIR*) were analyzed. The *SRR* and *CE* were calculated using Equations (1) and (2) [35]:

(1) $$SRR=\frac{C_{0}-C_{desalted}}{C_{0}}$$

(2) $$CE=\frac{zFQ_{desalted}(C_{0}-C_{desalted})}{NI}$$

Here, *C*_0_ denotes the initial feed concentration (mM) entering the channel, and *C_desalted_* represents the concentration of the desalted effluent (mM) exiting the channel. *z* represents the valence of the ion (1 for NaCl), *F* is the Faraday constant (96,485 C/mol), *N* is the number of cell pairs (*N* = 0.5 in this study), and *I* is the applied current (A). To further assess the energy efficiency, *EPIR* was defined as the specific energy consumed required to remove ions, as shown in Equation (3):

(3) $$EPIR=\frac{IV/Q_{desalted}}{zk_{B}T(C_{0}-C_{desalted})}$$

where *V* is the applied voltage (V), *k_B_* is the Boltzmann constant (1.38 × 10^−23^ J/K), and *T* is the absolute temperature (K).

**Figure 5:**
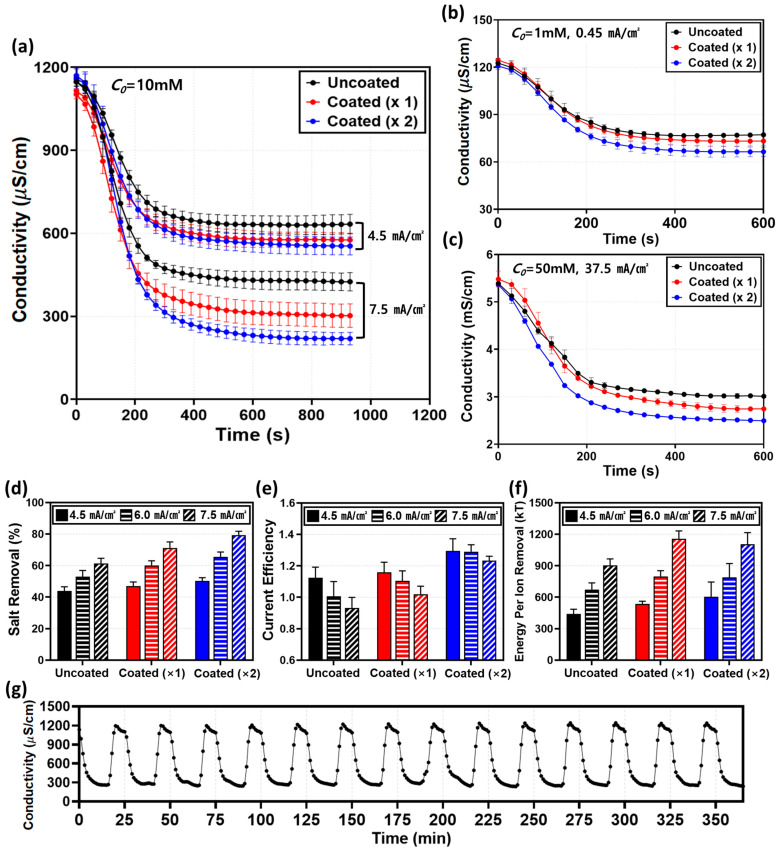
Quantitative evaluation of desalination performance, energy efficiency, and long-term stability in the micro-ICP system. (**a**–**c**) Time-dependent conductivity profiles of the desalted stream at varying initial concentrations (*C*_0_). The applied current densities were adjusted according to the concentration: (**a**) 4.5 and 7.5 mA/cm^2^ for *C*_0_ = 10 mM, (**b**) 0.45 mA/cm^2^ for *C*_0_ = 1 mM, and (**c**) 37.5 mA/cm^2^ for *C*_0_ = 50 mM. Across all regimes, the coated membranes exhibited faster ion depletion and lower final conductivity compared to the uncoated membrane. (**d**–**f**) Comparison of key performance metrics at *C*_0_ = 10 mM: (**d**) Salt Removal Ratio (*SRR*), (**e**) Current Efficiency (CE), and (**f**) Energy Per Ion Removal (*EPIR*). (**g**) Long-term stability test performed at *C*_0_ = 10 mM and 7.5 mA/cm^2^. The system underwent repetitive cycles comprising 16 min of desalination, 3 min of reverse-current regeneration, and 6 min of rest. The consistent conductivity drops over 15 cycles (approx. 6 h) demonstrate the mechanical and electrochemical durability of the coating layer. Error bars represent the standard deviation of independent measurements.

Before analyzing the specific mechanisms, we verified the robustness of the surface modification across a broad range of initial concentrations (*C*_0_ = 1, 10, and 50 mM), as shown in Figure 5a–c. Regardless of the bulk concentration, the coated membranes consistently exhibited higher *SRR* (Figure 5d) compared to the uncoated membrane, confirming that the induced electro-convective vortices effectively refresh the diffusion boundary layer in all regimes. Generally, in the over-limiting regime, *CE* tends to degrade due to the generation of parasitic currents from intensified water splitting [15,46,47,48]. The uncoated membrane in this study followed this typical behavior, exhibiting a decrease in *CE* to 0.93 as the current density increased to 7.5 mA/cm^2^ (Figure 5e). In stark contrast, despite the reduced LCD identified in Section 2.2, the coated membranes overcame this limitation, achieving a superior *CE* exceeding 100% (Coated ×2:1.23) and a high *SRR* of 79.4%. We attribute this performance enhancement to three distinct factors induced by the coating layer. First, minimization of parasitic current via water-splitting suppression. As visualized in the pH analysis (Figure 4b), the homogeneous Nafion layer physically sealed the catalytic sites on the heterogeneous surface. Unlike the uncoated membrane, where a significant portion of current was dissipated as leakage current due to water splitting, the coated membrane channeled the energy almost exclusively into salt transport [1]. Second, mitigation of current leakage and back-diffusion by the dense coating barrier. The dense Nafion layer observed in the SEM images (Figure 2) acts as a robust physical barrier that enhances perm-selectivity. This effectively inhibits the back-diffusion of ions from the concentrate to the diluate channel and prevents unwanted water transport [19], thereby maintaining high salt removal rates even in low-concentration regions. Third, maximization of mass transfer induced by intensified electro-convection. This is identified as the dominant factor. The macroscopic and powerful vortices observed in Section 2.3 (Figure 4a), triggered by the electrical nozzle effect of the coating, physically refreshed the diffusion boundary layer and induced vigorous hydrodynamic mixing [3,49]. As shown in Figure 5f, the *EPIR* values for both membranes increased sharply with current density, indicating that the operating current is the primary factor determining energy consumption. Although the coated membrane exhibited a slightly higher *EPIR* due to the additional resistance of the coating layer, the difference was marginal compared to the overall increasing trend driven by the current density. Unlike the uncoated membrane, which suffers from water splitting and reduced current efficiency in the over-limiting regime, the coated membrane maintains stable desalination performance. Consequently, the slight increase in energy consumption is accompanied by the significant improvement in process stability and salt removal efficiency. To further elucidate the correlation between material properties and performance, we compared the intrinsic characteristics of the coating and the substrate. Despite the lower intrinsic IEC of the Nafion coating (1.03–1.12 meq/g [50]) compared to that of the heterogeneous Ralex resin (1.88–2.34 meq/g [38]), the homogeneous ionomer layer effectively seals non-selective voids at the resin-binder interfaces. This structural sealing significantly enhances the apparent transference number (*t_app_*), as evidenced by the sharp rise in current efficiency (0.93 to 1.23) and suppressed co-ion leakage.

It is essential to discuss the theoretical upper limit of current efficiency in this system. According to Kwak et al. [35], in a CEM-CEM configuration where the diffusivity of anions (Cl^−^) exceeds that of cations (Na^+^), the theoretical maximum *CE* is determined by (1 + *D_Cl_-*/*D_Na+_*), yielding approximately 1.208. Our maximum experimental *CE* of ~1.23 slightly exceeds this theoretical value. However, considering the inherent sensitivity and potential deviations (typically 10–20%) in microfluidic platforms associated with in-line conductivity measurements at extremely low flow rates, this value should be interpreted as the system reaching its theoretical performance limit within the experimental margin of error. Rather than indicating an anomaly, this result confirms that the coated membrane effectively suppresses parasitic water splitting and back-diffusion, allowing the system to fully utilize the applied current up to its thermodynamic limit.

Finally, the long-term stability was confirmed over 15 consecutive cycles (approx. 6 h). As shown in Figure 5g, the system maintained consistent conductivity drops and rapid recovery without any performance degradation, demonstrating the mechanical and electrochemical durability of the coating layer against the prolonged hydrodynamic shear stress generated by electro-convection. Conclusively, these results empirically demonstrate that in microfluidic ICP systems, the overall process efficiency is determined not merely by low membrane resistance, but by the synergy of suppressing water splitting, preventing leakage, and enhancing electro-convective mixing.

## 3. Materials and Methods

The experimental procedure of this study consists of three main stages: surface modification of heterogeneous membranes (Membrane Preparation), fabrication of the microfluidic device (Device Fabrication), and the experimental setup for visualization and performance evaluation (Experimental Setup and Methods).

### 3.1. Membrane Preparation

**Figure 6:**
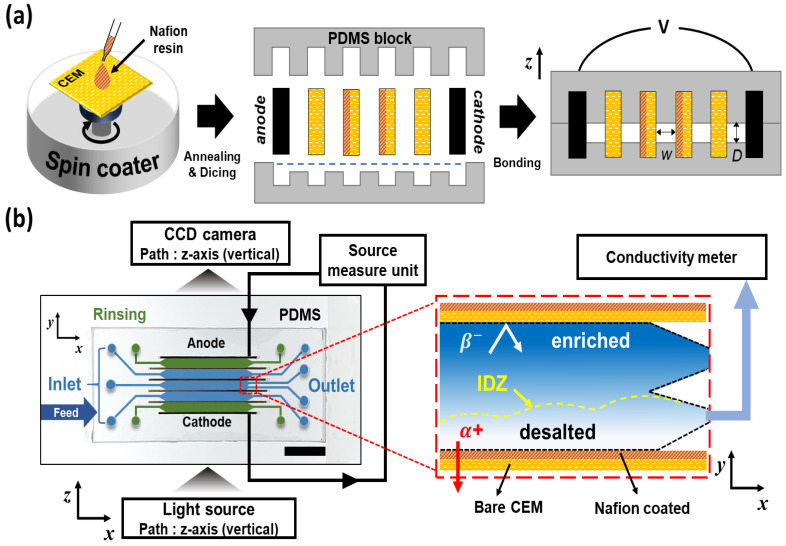
Schematic illustration of the device fabrication process and the experimental setup for ICP-based desalination. (**a**) Fabrication procedure of the microfluidic device. The heterogeneous CEM substrate is surface-modified via spin-coating with Nafion resin, followed by annealing and dicing into specific dimensions. The prepared membranes are inserted into the slots of the PDMS block, and the device is sealed. The cross-sectional view (**right**) illustrates the internal channel geometry, where *W* and *D* denote the inter-membrane distance (2 mm) and channel depth (0.2 mm), respectively. The channel length (*L*) was fixed at 20 mm. (**b**) Experimental setup for real-time visualization and electrochemical performance evaluation. The integrated system comprises a source measure unit (SMU) for applying electrical potential, an inverted fluorescence microscope (equipped with a CCD camera and light source) for capturing electro-hydrodynamic vortex dynamics, and a flow-through conductivity meter for monitoring desalination efficiency at the outlet. The enlarged inset (red box) depicts the ion transport mechanism driven by Ion Concentration Polarization (ICP). Under the applied electric field, cations (*α*^+^) selectively migrate through the CEMs, leading to the formation of an expansion of the Ion Depletion Zone (IDZ) and a desalted stream, while the migration of anions (*β*^−^) is restricted, creating an enriched zone.

A commercial heterogeneous cation exchange membrane (CEM), Ralex CMHPP (MEGA, Prague, Czech Republic), was selected as the base membrane [38]. For surface modification, a perfluorinated sulfonated polymer solution, Nafion D2020 dispersion (20 wt%, Chemours, Wilmington, DE, USA), was utilized as the coating material. The pretreatment and coating processes were as follows: First, the CEM was cut into 3 cm × 3 cm pieces. The Nafion solution was then applied onto the base membrane using a spin coater (SF-100A, Rhabdos, Seoul, Republic of Korea). To ensure the formation of a uniform coating layer, spin coating was performed at 1000 rpm for 30 s. The thickness of the coating layer was controlled by adjusting the number of coating cycles to one and two times. After the coating process, thermal annealing was conducted on a hot plate at 65 °C for 30 min to completely remove residual solvents and enhance the physical adhesion between the coating layer and the base membrane. The schematic of the overall membrane fabrication process is illustrated in Figure 6a. The prepared membranes were designated as Uncoated (bare), Coated (×1) (single coating), and Coated (×2) (double coating). The surface wettability of the membranes was evaluated using the static sessile drop method. Prior to measurement, all membrane samples were immersed in deionized (DI) water for over 12 h to ensure a fully hydrated state. A DI water droplet with a volume of approximately 8–10 μL was deposited onto the membrane surface, and the contact angle was determined using the tangent method within 20 s to minimize errors caused by evaporation or absorption. To ensure statistical reliability, measurements were repeated at least five times at random locations for each sample, and the average values were reported. The thickness of the coating layer was determined directly from the cross-sectional SEM images. The basic physicochemical and electrochemical properties of each membrane are summarized in Table 1.

### 3.2. Device Fabrication

A microfluidic device was fabricated to observe the electro-convective vortices generated within the electromembrane system and to measure the corresponding electrical signals. The device fabrication followed the method for polydimethylsiloxane (PDMS)-based devices established in previous studies [34,35,36]. Specifically, top and bottom PDMS blocks were prepared by casting PDMS prepolymer into master molds produced via a 3D printer, followed by curing and peeling. Figure 6b shows the schematic of the PDMS device and the ion transport mechanism within the main channel. The fabricated device consists of two electrodes, four ion exchange membranes (IEMs), three main channels, and two electrode rinsing channels. It was designed with an Ion Concentration Polarization (ICP)-based desalination structure using only CEMs [35], rather than a conventional electrodialysis stack alternating between anion and cation exchange membranes. The device features five inlets and six outlets, where the latter utilize a Y-shaped bifurcation monolithically integrated via the 3D-printed mold [35] to physically separate the enriched and desalted streams. Carbon paper (Spectracarb 2050A-1550; Fuel Cell Store, Bryan, TX, USA) was inserted at the outermost parts of the device to serve as electrodes. The depth (*D*) and width (inter-membrane distance, *W*) of all channels were fixed at 0.2 mm and 2 mm, respectively, with a channel length (*L*) of 20 mm. The coated and uncoated membranes were cut into 3 cm × 0.5 cm pieces to fit the device slots; the coated membrane was inserted into the center of the main channel, while the uncoated membranes were placed in the outer slots. Subsequently, the top and bottom PDMS blocks were permanently bonded via oxygen plasma treatment. Although the membranes do not chemically bond with the PDMS, a hermetic seal was achieved by utilizing the swelling phenomenon of the membranes upon electrolyte injection. The slots were designed with a slight clearance relative to the dry membranes to facilitate assembly. Once hydrated, the membranes expanded against the elastic PDMS walls, creating a self-sealing effect that effectively prevented fluid leakage. To ensure stable hydration and establish the hermetic seal, a 10 mM NaCl solution was injected into the channel immediately after assembly, and the system was maintained for 24 h. This in situ equilibration step not only facilitated the physical swelling of the membranes against the PDMS walls but also ensured that both the Ralex substrate and the Nafion coating layer were fully converted to the Na^+^ ionic form prior to the experiments.

### 3.3. Experimental Setup and Methods

Experiments were comprehensively conducted under two distinct operating modes: constant voltage and constant current conditions. The constant voltage mode was utilized to visually observe the micro-scale electro-convective vortices at the membrane interface and to analyze the current response characteristics. Conversely, the constant current mode was employed to quantitatively evaluate water-splitting reactions and desalination efficiency under practical operating conditions. Figure 6b illustrates the overall experimental setup. A precision syringe pump (Fusion 200-X, Chemyx, Stafford, TX, USA) was used to control fluid flow. A 10 mM sodium chloride (NaCl) solution was injected into the main channel, while a 5 mM sodium sulfate (Na_2_SO_4_) solution was supplied to the electrode rinsing channels to remove electrode reaction byproducts and maintain electrical conductivity. All solutions were prepared using ultra-pure deionized water with a resistivity of 18 MΩ·cm and were degassed prior to use to eliminate microbubbles. Unless otherwise specified, the flow velocity was fixed at 1 mm/s. To minimize thermal fluctuations and ensure the reliability of conductivity measurements, all experiments were carried out in a temperature-controlled laboratory maintained at 25 ± 1 °C. Before data acquisition, the system was operated for approximately 10 min to ensure hydrodynamic stabilization. A high-precision source meter (2460 Source Meter, Keithley, Beaverton, OR, USA) was employed for both power supply and signal measurement. Visualization experiments under constant voltage conditions were performed in a quiescent state (no flow) to eliminate external flow interference, with applied voltages of 15 V and 20 V corresponding to the over-limiting regime. To trace the fluid motion, a negatively charged fluorescent dye of 19.09 μM (Alexa Fluor 488 Triethylammonium, Thermo Fisher Scientific Korea, Seoul, Republic of Korea) was mixed into the NaCl solution in the main channel. The initiation, growth, and merging processes of electro-convective vortices at the membrane interface were recorded in real-time using an inverted fluorescence microscope equipped with a high-speed camera. For Current-Voltage (I-V) curve characterization, the flow velocity was set to 1 mm/s. The voltage was swept from 0 V to 12 V in 0.3 V increments. At each voltage step, the duration was set to 30 s to allow the current to reach a steady state, minimizing transient effects before recording the average current value. Under constant current conditions, experiments focused on the in-depth evaluation of water splitting and desalination performance. First, pH visualization was performed at a flow velocity of 1 mm/s with an applied current of 300 μA (corresponding to a current density, *J* = 7.5 mA/cm^2^). By incorporating a universal pH indicator into the NaCl solution and monitoring local color changes at the membrane interface, the occurrence of water splitting induced by intensified concentration polarization was visually verified. Desalination experiments were carried out at a constant flow velocity of 1 mm/s under two current conditions (180 μA [*J* = 4.5 mA/cm^2^] and 300 μA [*J* = 7.5 mA/cm^2^]). The conductivity of the desalted stream downstream was measured in real-time using a flow-thru conductivity electrode (16–900, Microelectrodes, Bedford, NH, USA) connected to a benchtop meter (Orion Star A215, Thermo Fisher Scientific Korea, Seoul, Republic of Korea). Data were recorded after the conductivity readings stabilized, using the average value over a 5 min period to ensure reliability. Based on these measurements, the salt removal ratio (*SRR*) and current efficiency (*CE*) were calculated. Additionally, chronopotentiometry was employed to precisely measure the variations in Ohmic resistance relative to coating thickness and to determine the transition time (τ), which indicates the rapid voltage rise due to concentration polarization [51].

## 4. Conclusions

In this study, we proposed a surface modification strategy to upgrade low-cost heterogeneous ion-exchange membranes (IEMs) and visually elucidated the correlation between surface coating, electro-hydrodynamic (EHD) behavior, and actual desalination performance using a microfluidic platform. Our results demonstrate that the Nafion coating layer effectively planarized the rough surface defects of the heterogeneous membrane and established a homogeneous conductive interface. The primary novelty of this work lies in the direct visualization of the mechanisms driving performance enhancement. We empirically proved that the coating structure induces an electrical nozzle effect via localized electric field focusing, acting as a critical trigger for macroscopic electro-convective vortices. Furthermore, pH visualization verified that the coating layer physically seals catalytic sites, significantly suppressing water splitting reactions. Consequently, in the high-current regime (300 µA), the coated membranes achieved superior performance, recording a current efficiency of 1.23 (an increase of ~32%) and a salt removal ratio of 79.4% (an increase of ~18%) compared to bare heterogeneous membranes. We attribute this performance enhancement to three distinct factors induced by the coating layer: (1) Elimination of parasitic currents: As verified by visualization, the homogeneous coating suppressed water splitting, minimizing current loss. (2) Prevention of leakage and back-diffusion: The dense ionomer barrier enhanced perm-selectivity by inhibiting ionic leakage and back-diffusion. (3) Maximization of mass transfer: Most critically, the visualized macroscopic vortices physically refreshed the diffusion boundary layer and formed an extended instability region, accelerating ion transport. It is worth noting that the confined swelling pressure contributing to these effects is characteristic of the microfluidic configuration with rigid clamping. In macroscopic electrodialysis stacks with larger channel dimensions, the mechanical boundary conditions may differ, which could be an interesting subject for future comparative studies.

In conclusion, by utilizing micro-scale visualization to unambiguously identify the mechanisms of vortex enhancement and water-splitting suppression, this study establishes that optimizing hydrodynamic instability is a more dominant design factor for high-efficiency desalination than merely minimizing membrane resistance.

## Figures and Tables

**Table 1:** Physicochemical properties of the uncoated and Nafion-coated heterogeneous ion-exchange membranes, including thickness variations in dry/wet states and surface wettability.

CEM Type	Nafion Coating Thickness ^(a)^ (µm)	Total Membrane Thickness ^(b)^ (Dry, µm)	Total Membrane Thickness ^(b)^ (Wet, µm)	Contact Angle (°)
Uncoated (Ralex CMHPP)	-	450 ± 10	590 ± 15	71.0 ± 4.1
Nafion Coated (×1)	8	460 ± 10	600 ± 10	59.0 ± 3.1
Nafion Coated (×2)	10	460 ± 10	610 ± 10	58.0 ± 3.7

^(a)^ Determined from cross-sectional SEM images (dry state). ^(b)^ Measured using a digital micrometer (resolution limit: 10 µm).

## Data Availability

Data are contained in this article.
